# Value pluralism in research integrity

**DOI:** 10.1186/s41073-019-0076-4

**Published:** 2019-08-22

**Authors:** Rik Peels, Jeroen de Ridder, Tamarinde Haven, Lex Bouter

**Affiliations:** 1 Philosophy Department, Vrije Universiteit Amsterdam, De Boelelaan 1105, 1081 HV Amsterdam, The Netherlands; 2 Department of Epidemiology and Biostatistics, Amsterdam UMC, location VUmc, De Boelelaan 1089a, 1081 HV Amsterdam, The Netherlands

**Keywords:** Code of conduct, Epistemology, Ethics, Pluralism, Principle, Value, Virtue

## Abstract

Both scientists and society at large have rightfully become increasingly concerned about research integrity in recent decades. In response, codes of conduct for research have been developed and elaborated. We show that these codes contain substantial pluralism. First, there is *metaphysical pluralism* in that codes include values, norms, and virtues. Second, there is *axiological pluralism*, because there are different categories of values, norms, and virtues: epistemic, moral, professional, social, and legal. Within and between these different categories, norms can be incommensurable or incompatible. Codes of conduct typically do not specify how to handle situations where different norms pull in different directions. We review some attempts to develop an ordering of different sorts of norm violations based on a common measure for their seriousness. We argue that they all fail to give adequate guidance for resolving cases of incommensurable and conflicting norms. We conclude that value pluralism is inherent to codes of conduct in research integrity. The application of codes needs careful reasoning and judgment together with an intellectually humble attitude that acknowledges the inevitability of value pluralism.

## Introduction

This paper starts the project of developing an axiological framework for classifying and comparing the elements of codes of conduct for research integrity. We will investigate the contents of a set of codes systematically, in order to bring more theoretical clarity to them and to develop a framework in which the notion of research integrity can be articulated with greater conceptual clarity and precision. We will see that there is irreducible *value pluralism* in research integrity: there is a variety of different kinds of values underlying codes of conduct that cannot be reduced to each other and that give rise to a plurality of virtues and norms, some of which are either incommensurable or in conflict with each other. Thus, when we refer to “value pluralism,” we have in mind the plurality of values that underlies research codes, but also mean to include the plurality of norms and virtues that this plurality of values gives rise to (below, we will say more about how values relate to norms and virtues).

We will not attempt to give an all-encompassing characterization of research integrity here—that would be overly ambitious—but we will specify several ingredients that any future satisfactory characterization should include. Our study is primarily conceptual and not qualitative empirical.^1^ Hence, we refer to the codes we selected to illustrate our conceptual points. We do not aim to defend any particular set of values or norms that can provide a normative foundation for existing codes of research integrity.^2^ Rather, we argue that any such foundation must be pluralistic because value pluralism in research integrity is inevitable.

With an eye to representativeness, we have selected codes of conduct on the basis of three criteria: geographical diversity, age (the more recent, the better), and independent origins. Our sample includes the following: the 2017 *European Code of Conduct for Research Integrity*, also referred to as the “ALLEA code” (for Europe) [1, 37]; the 2011 *Directives on Research Integrity Brazil* by the Brazilian Council for Scientific and Technological Development [9], as well as the 2012 complementary code of conduct *FAPESP—Code of Good Scientific Practice* by the Sao Paulo Research Foundation (for South-America) [48]; the 2014 *Guidelines for Responding to Misconduct in Research* by the Ministry of Education, Culture, Sports, Science and Technology in Japan (for Asia) [23]; the 2016 *Australian Code for the responsible Conduct of Research* by Universities Australia, Australian Government, National Health and Medical Research Council, and Australian Research Council (for Australia) [3]; the 2004 *Guidelines for Researchers and Ethics Review of Committees in Zimbabwe* by the Medical Research Council of Zimbabwe (for Africa) [22]; and finally, the 2013 *Code of Conduct* by the National Research Council Canada (for North-America) [36].^3^

The rest of the article is structured as follows. First, we provide some background to motivate the project of the paper (the “Background” section). We then introduce distinctions between several kinds of things one finds in codes of conduct on research integrity, especially values, virtues, and norms (the “Metaphysical pluralism: values, norms, and virtues” section). Subsequently, we show that norms can be classified in at least five different categories: epistemic, moral, professional, social, and legal (the “Axiological pluralism: five categories of normativity” section). After that, we show two ways in which value pluralism manifests itself in codes of conduct: incommensurable norms (the “Axiological pluralism 1: Incommensurable norms from different categories” section) and incompatible norms (the “Axiological pluralism 2: Conflicting norms from the same category” section). In doing so, we shall largely focus on epistemic and moral norms. We then consider three attempts to overcome incommensurability and incompatibility and argue that in spite of their use, they do not solve the problem of value pluralism in research integrity. Finally, we draw a few conclusions and formulate recommendations for future work on this (the “Overcoming pluralism?” section).

## Background

Both scientists and society at large have rightfully become increasingly concerned about research integrity in recent decades. Among the reasons for this are various high-profile cases of research misconduct, the apparently high prevalence of questionable research practices in some academic disciplines, and growing concerns over sloppy science and research waste [8, 10, 17, 24, 32, 33].

Part of the response to these concerns has been the development and further elaboration of codes of conduct for research integrity. These codes provide core values, rules, and principles that seek to codify what it is to conduct scientific research responsibly and to avoid fraud and questionable research practices. They are typically drawn up in close collaboration with scientists, by scientific institutions such as disciplinary associations, offices for research integrity, funding agencies, and national and international academies of science. In Europe alone, there are more than 40 national codes of conduct for research integrity [19, 20].

These codes of conduct are a mixed bag of mixed bags, in the following sense. First, there are many codes with significantly different contents. Moreover, the terminology and classification systems used across different codes vary: something that is described as a value in one code might show up as a principle or a responsibility in another. Second, even single codes typically use a wide variety of terms: a cursory glance reveals that some are organized around core “values,” others contain long lists of “rules” or “principles,” sometimes accompanied by more concrete “applications,” yet others talk about “duties” or “responsibilities,” and we find “best practices” as well as various combinations of these items.

All by itself, this does not show that there is anything wrong with current approaches to research integrity. Just as there are many roads to Rome, there may be many roads to capturing what responsible conduct of research is. Nonetheless, it would be helpful if there were a more encompassing systematic *axiological framework* that identifies and classifies the elements of research integrity and responsible conduct of research. There are several reasons for this. *First*, the absence of such a framework might give the false impression that the notions of research integrity and responsible conduct of research lack clear meaning and that their definitions are up for grabs. *Second*, a framework can provide conceptual clarity and systematicity that can be helpful in carrying out future research on research integrity, but also in disseminating, implementing, and communicating these codes. *Third*, a general framework can bring out overlap or outright duplication in codes of conduct, thus avoiding unnecessary complications and confusions. *Fourth*, it might identify blind spots in codes: values, norms, academic misbehaviors, or questionable research practices that may have been overlooked. *Fifth*, a framework can function as a means for recognizing and navigating trade-offs in responsible conduct of research transparently. Philosophers of science have long recognized that the values and norms that operate in science can pull in different directions [15, 30, 31]. *Sixth*, a framework can be a step towards a more systematic and fine-grained comparison and assessment of the gravity of questionable research practices and misconduct. Such comparisons and assessments are important for (i) identifying and developing the most promising and effective interventions for improving scientific practice, and (ii) avoiding arbitrariness and capriciousness in sanctioning breaches of integrity. Breaches are typically evaluated on the basis of codes of conduct that are specific to the institute, funding agency, discipline, or country of the case. However, without a general framework to compare and weigh different breaches of misconduct, organizations run the risk of applying sanctions more or less arbitrarily. To be clear, a framework by itself is not enough to make the required comparisons and assessments, but it can help to see more clearly what values, norms, etc. should go into them.

## Metaphysical pluralism: values, norms, and virtues

Before we look at actual codes of conduct in more detail, it is helpful first to ask a more general question: what *sorts* of things are in these codes? As noted above, a quick glance reveals a significant variety in the sorts of things that codes of conduct are organized around and describe: (i) *values*, (ii) *norms*, *rules*, *standards*, or *principles* (we will use these terms synonymously), (iii) *virtues*, (iv) *responsibilities* or *duties*, and (v) *good (or bad) practices*. Let us first get a better grip on what these things are.

### Values

Values are universals, more specifically good-making properties that actions, events, objects, or persons can have. They are what makes something have worth and be desirable.^4^ For example, to say that truth is a value is to say that it is desirable for claims, theories, or other truth-apt items to be true or, more precisely, that it is desirable for people to believe and say things that are true. Even though most codes do not use the term “value” explicitly, we find various examples of values in them. For instance:

- Impact, accountability, leadership, integrity, and collaboration (Canada Policy, p. 3);
- Moral leadership, honesty, integrity, rigor, transparency, fairness, respect, recognition, and accountability (Australian Code, pp. 1–2);
- Knowledge and trust (Guidelines from Japan, pp. 1–2);
- Intellectual honesty, objectivity and impartiality, truthfulness, and fairness and responsibility (FAPESP, Brazil, p. 21);^5^
- Respect for autonomy and protection of persons with impaired or diminished autonomy (Guidelines from Zimbabwe, p. 3).

### Norms

Norms (principles, rules, directives, standards, or guidelines) are statements that describe or prescribe desirable, required, or optimal behavior for researchers or that describe, proscribe, or discourage undesirable, forbidden, or suboptimal behavior. Norms say how people ought, and ought not, to conduct themselves. There are norms at different levels of generality and abstraction, ranging from the very general (“do the right thing”) to the specific and concrete (“use Chicago style in preparing your manuscript for this journal”).^6^ They can also differ in their range of application. Some norms apply to everyone, others only to specific subgroups or individuals, such as, in our case, scientists and scholars. The bulk of codes of research integrity consists of norms. Here are a few examples of general norms that apply to all academic disciplines:

- Use the unpublished work of other researchers only with permission and due acknowledgement and use published work of other researchers with due acknowledgement (Canada Policy, p. 4);
- Researchers must precisely and completely record the data and information collected, the procedures utilized, and any partial results obtained during the course of a research study (Directives from Brazil, p. 24).

Some norms only apply to some academic fields because of their content. For instance, rules about data management are irrelevant to fields using very little or no empirical data, such as mathematics or philosophy:

- Researchers, research institutions, and organizations ensure appropriate stewardship and curation of all data and research materials, including unpublished ones, with secure preservation for a reasonable period (ALLEA, p. 6).

Obviously, norms such as the following are only relevant to fields that do experiments involving animals:

- Ensure replacement, reduction, and refinement will be considered at all stages of research involving animals. Act to minimize the impacts on animals used in research and in so doing support the welfare and wellbeing of these animals (Australian Code, p. 4);
- Appropriate caution must be exercised in the conduct of research which may affect the environment, and the welfare of animals used for research must be respected (Guidelines from Zimbabwe, p. 18).

Various codes also list examples of *violations* of academic integrity: research misconduct and unacceptable practices (Directives from Brazil, pp. 31–32). Although these are not explicitly formulated as norms, they can be straightforwardly interpreted as such: avoid misconduct and do not engage in other unacceptable practices. Misconduct is traditionally defined as fabrication, falsification, and plagiarism (Directives from Brazil, p. 31). Another example of an unacceptable practice is:

- Manipulating authorship or denigrating the role of other researchers in publications (ALLEA, p. 8).

### Virtues

Virtues are “qualities that make a person excellent” ([5], p. 2), that is, character traits or behavioral dispositions that individuals can have and that have positive value. They can be subdivided in moral and intellectual virtues. Moral virtues are constitutive of and conducive to a morally upstanding and flourishing life (cf. [26]), while “intellectual virtues are characteristics that promote intellectual flourishing, or which make for an excellent cognizer” ([49]; cf. also [14]).^7^

These broad characterizations are fleshed out in different ways by different virtue theorists. Some maintain that intellectual virtues are mere reliable cognitive processes [21, 46, 47], while others construe intellectual virtues more robustly, as involving proper affective and volitional states, in addition to cognitive aspects [4, 42, 50]. We can ignore these differences here and work with a broad inclusive notion of virtue that covers both the “thin” and “thick” construals of virtue.

Although no code of conduct included here mentions virtues explicitly, they do contain many items that are most naturally understood as character traits or good-making qualities of individuals or groups. Here are some examples:

- Reliability, honesty, respect, and accountability (ALLEA, p. 4);^8^
- Intellectual honesty, objectivity and impartiality, truthfulness, and fairness and responsibility (Directives from Brazil, p. 21).

Note that some items on this list, such as reliability and honesty, can be understood generally as a *value*—some good-making property that actions, studies, people, events, or instruments can have—or as a *virtue*, that is, as a moral or intellectual character trait of researchers or perhaps even teams and organizations. This explains why several items on the above list can also be found under “values.” In fact, it follows from the above characterization of values that virtues are, properly speaking, a subset of values. They are, after all, features that make something (in this case, persons) good. The reason to discuss them separately is that they are typically singled out as an especially relevant class of values, since responsible conduct of research directly concerns the behavior and dispositions of researchers.

### Responsibilities, duties, and practices

Codes further contain responsibilities, duties, and practices. Responsibilities relate persons to actions or their consequences and make normative appraisal of the person in question possible. If it is your responsibility to enter data carefully or to see to it that it gets done carefully, you are not doing a good job if you do not spend any time in the lab. Duties are similar; they also relate agents to actions or their consequences, by stipulating that there are actions that an agent is obligated or required to do, or consequences she ought to bring about.

The important thing to see is that responsibility and duty talk can easily be translated into norms talk. If it is your responsibility to do X, then the norm “do X” applies to you. If it is your duty to avoid Y, that boils down to a rule that you ought to avoid Y.

Some codes of conduct further describe good, questionable, or unacceptable research practices. We can think of these as ways of conducting, organizing, and evaluating research that are desirable, undesirable, or even precluded, because they exemplify a good or ideal way of doing things, or rather violate it. Talk about practices, too, can easily be translated into talk about norms. We will, therefore, not treat responsibilities, duties, and practices separately but take them into account in our discussion on norms.

This leaves us with three kinds of items that make up the bulk of codes for responsible conduct of research: *values*, *norms*, and *virtues*. These things are generally thought to be metaphysically different.^9^ Appreciating this matters for reasons of analytical clarity, but arguably also has practical import. First, in so far as codes emphasize *norms*, their implementation and use might promote rule-following and communication about procedures, protocols, etc. An emphasis on *virtues*, on the other hand, more easily translates to things like mentoring, character-building, and interventions to create a culture of research integrity. Second, failing to appreciate the differences between values, norms, and virtues can lead to confusion in assessing behavior in the light of a code of conduct. For example, one cannot straightforwardly compare an abstract value such as truth with a specific intellectual character trait, such as intellectual thoroughness. Failure to appreciate the difference between virtues and norms might lead one miss the insights that, sometimes, following all the norms to the letter can still fall short of intellectually virtuous behavior and that virtue can sometimes requires one to flout a norm.

Saying that values, norms, and virtues are metaphysically distinct is not to deny that they bear relations to each other. Values are typically fairly abstract qualities. Norms give concrete prescriptions for how we can realize or promote values. For example, if honesty is a value, then researchers should refrain from overstating their conclusions and from downplaying the study limitations. We can thus say that norms derive from one or more values and that values give rise to norms. Virtues, in turn, are also conducive to the realization of more abstract values. If researchers possess virtues such as carefulness, thoroughness, and open-mindedness, this will be conducive to the acquisition of knowledge. Virtues are usually also fairly abstract and can be made concrete by specifying characteristic rules that follow from them. Although acting virtuously will typically mean that researchers follow rules for responsible conduct, it might occasionally mean that they not follow them to the letter, because possessing virtues also involves sensitivity to the limits of rules, to the different ways in which they might be interpreted, and to their spirit rather than their letter. So the first kind of pluralism in scientific codes of conduct is of a *metaphysical* sort: codes describe different kinds of things.

In what follows, we shall focus on norms. This is because they are more concrete: they prescribe or prohibit specific behavior. As a result of that, potential conflict, tension, or incommensurability between them is more perspicuous than with the more abstract and vague categories of values and virtues. This makes norms highly suited to bring out a second kind of pluralism in codes of conduct, stemming from different categories of values, virtues, and norms.

## Axiological pluralism: five categories of normativity

The second form of pluralism in codes of conduct is *axiological*. It follows from the fact that there are different *categories* of norms. This is because there are different categories of normativity and normative appraisal: epistemic, moral, professional, social, and legal. “Do not lie” is a moral norm, but not all lying is prohibited by a legal norm and in some situations social norms in fact call for lying. There is overlap between these normative categories. Legal norms typically align with and codify moral and social norms. Sometimes, professional norms are formally encoded in legal norms. Since scientific research is in the business of truth-finding, many professional norms for research are indirectly or directly related to epistemic norms. Moreover, specific behaviors and practices may be desirable or undesirable from different normative perspectives. Good mentoring, for example, is desirable from a moral, social, and professional perspective. This sometimes makes it difficult to peg norms into a single category. Nonetheless, other rules clearly do belong in one category: it is a professional norm, for instance, to use an accepted bibliographical style for referring to literature, but doing so has little or no relevance from other normative perspectives. Codes of conduct contain items from all five categories, as we will now show.^10^

1. *Epistemic* norms have to do with what is desirable or required from the perspective of acquiring and maintaining true beliefs, knowledge, and understanding, and avoiding false beliefs, ignorance, and unsupported or unjustified belief. Since the goals of science are first and foremost epistemic, we find many examples of epistemic norms in codes of conduct.

- Do not misinterpret data to obtain desired results, where this includes inappropriate use of statistical methods (Canada Policy, p. 6);
- Researchers report their results in a way that is compatible with the standards of the discipline and, where applicable, can be *verified* and *reproduced.* (ALLEA, p. 7, emphasis added).

(2) *Moral* norms are norms that pertain to the treatment and wellbeing of individuals, animals, and groups, as well as the flourishing of nature (flora and fauna). Examples are as follows:

- Fellow researchers will be treated fairly and with respect (Australian Code, p. 2);
- Respect for animals must underpin all decisions and actions related to the care and use of animals in research (Australian Code, p. 2).

(3) *Professional* norms are norms the violation of which does not necessarily lead to epistemic or moral harms, but to behavior that is nonetheless unacceptable by the standards of the profession. Scholarly and scientific research has its implicit and explicit conventions and rules that regulate what it is to do one’s work well. Examples include the following:

- Establish good governance and management practices for responsible research conduct (Australian Code, p. 3);
- The inclusion of authors in the manuscript should be discussed before starting the collaboration and should be based on established guidelines, such as those from the *International Committee of Medical Journal Editors* (Directives from Brazil, p. 2);
- Any researcher that publishes a scientific work that is identical or substantially similar to a work already published should clearly and prominently cite the first publication in the text of the work (Directives from Brazil, p. 23).
- All partners in research collaborations take responsibility for the integrity of the research (ALLEA, p. 6).

It is not always easy to distinguish professional norms from norms in other categories. First, the goal of science is the pursuit of truth and knowledge. Hence, many professional norms of science bear directly or indirectly on that goal and are therefore also partly epistemic. Norms like “provide accurate source references” or “keep the bibliography at a reasonable size” make it easier to assess the support for claims presented in an article. Arguably, this is conducive to knowledge production. Second, as professional norms become more widely accepted and entrenched, it becomes *morally* unacceptable to violate them. It may have been acceptable at one time to “mentor” Ph.D. students by throwing them in at the deep end only to wait if they would resurface again, but nowadays this is—or should be—morally unacceptable. Professional norms for good mentorship have become more demanding.

(4) *Social* norms are norms that are relevant to the wellbeing and flourishing of the society in which the research is carried out. Here are examples:

- Aboriginal and Torres Strait Islander peoples and communities will be consulted and involved in decisions about research and will be informed about research outcomes and benefits (Australian Code, p. 2);
- Researchers have due regard for the health, safety, and welfare of the community, of collaborators, and of others connected with their research (ALLEA, p.7).

(5) *Legal* norms are norms that can be found in official law or jurisprudence. Obviously, legal norms are often inspired by what legislative authorities take to be epistemic, moral, social, and professional norms. Yet, there may be reasons to put in place legislation even if there are corresponding epistemic, moral, social, and professional norms—say, for the sake of deterrence. Here are examples:

- Research will comply with all relevant legislation and governmental and institutional policies and guidelines (Australian Code, p. 2);
- One of the responsibilities of researchers is observing laws and relevant regulations in conducting research (Guidelines form Japan, p. 29);
- Whether or not, in the country in which the prospective subject is invited to participate in research, the right to compensation is legally guaranteed (Directives from Zimbabwe, p. 23);
- Under the Access to Information and Privacy (ATIP) Acts, any personal information or written information on the conduct and conclusions of the research integrity process can only be shared within the limits of the legislation or if the persons involved agree (Canada Policy, p. 11).

We take it that these five categories cover virtually all aspects of research integrity.

## Axiological pluralism 1: Incommensurable norms from different categories

We have identified two sorts of pluralism in codes of conduct for research integrity: metaphysical and axiological. That is to say, we have seen that codes of conduct contain metaphysically different things: values, norms, and virtues. While there are relations between them, they are not the same. We have also seen that there are axiologically different categories of norms: epistemic, moral, professional, social, and legal.

In this and the next two sections, we argue that this pluralism is inevitable, and not a contingent feature of the way codes of conduct are currently organized. Any viable code of conduct for research integrity will display substantial pluralism. In this section, we discuss two examples of *incommensurability* between norms from different categories. When norms are incommensurable, they *cannot be compared to one another or put in one single ordering because they do not map onto a single common scale*.^11^ In the next section, we will give examples of *conflicting* (and potentially incommensurable) norms within the same categories. Conflicts between norms show the need for trade-offs in interpreting what research integrity means in situations where two or more conflicting norms apply.^12^

### Example 1

The ALLEA code contains a variety of epistemic norms for openness and transparency that are aimed at wide distribution of data and knowledge. Here are three instances:

- Researchers, research institutions, and organizations ensure access to data is as open as possible, as closed as necessary, and where appropriate in line with the FAIR Principles (Findable, Accessible, Interoperable, and Re-usable) for data management (p. 6);
- Researchers, research institutions, and organizations acknowledge data as legitimate and citable products of research (p. 6);
- Authors ensure that their work is made available to colleagues in a timely, open, transparent, and accurate manner, unless otherwise agreed, and are honest in their communication to the general public and in traditional and social media (p. 7).

These *epistemic* norms can point in another direction than *professional* norms about joint work and agreements with other (sometimes commercial) parties about (temporary) secrecy or privacy, as they are mentioned in sections 2.3 and 2.6 of ALLEA:

- Researchers publish results and interpretations of research in an open, honest, transparent, and accurate manner, and respect confidentiality of data or findings when legitimately required to do so (p. 6).
- All partners formally agree at the start of their collaboration on expectations and standards concerning research integrity, on the laws and regulations that will apply, on protection of the intellectual property of collaborators, and on procedures for handling conflicts and possible cases of misconduct (pp. 6–7);

From a *purely* epistemic perspective, disclosing everything, complete openness, and full transparency would be optimal, as it would produce more and more widely shared knowledge. From a *purely* professional (and legal) perspective, on the other hand, it is arguably paramount to respect agreements, mutual expectations, and contracts, no matter how much non-disclosure and secrecy they entail. So epistemic norms and professional norms pull in different directions here. It should be noted that the formulations of the specific norms above already have qualifications built into them to navigate tensions between openness and closedness, between sharing and not-sharing: e.g., “as open as possible,” “as closed as necessary,” and work is to be made available “unless otherwise agreed.” This reinforces our earlier point that the concrete norms in codes of conduct often cannot be placed in one of our five categories exclusively.

The important point for now is that it is difficult to adjudicate the tension between openness and non-disclosure because it derives from two incommensurable values underlying these norms. It is unclear how one can compare the epistemic value of openness with the professional/legal value of honoring agreements and contracts. Or, better, it seems that they cannot be compared at all. To see this, ask yourself how one might go about making a principled argument that, say, openness is more important than respecting a non-sharing clause in an agreement with a sponsor. It is not as if we have a measuring stick to determine and compare the worth of openness and that of secrecy, nor is there a more general value or perspective under which both might be subsumed.^13^

Hence, it is unclear that there is a uniquely correct answer as to which of the two should prevail in a given scenario, because the relevant epistemic and professional/legal values behind the norms do not map onto one common scale. Of course, one can decide to let one or the other prevail in cases of conflict, but that does not remove the incommensurability. Scholars in research integrity ought to be aware of such incommensurability, since it enables us to appreciate that others might make different choices that may well be equally reasonable. Incommensurable norms can lead to different reasonable verdicts in particular cases.

### Example 2

Epistemic norms that aim at maximizing the amount of true beliefs produced by pursuing original and innovative projects can lead to different verdicts than professional norms that aim at realistic goals and clear deliverables. The Code of Conduct from Canada, for instance, says that researchers should be innovative and that managed risk-taking should be encouraged (p. 4). At the same time, it says that realistic goals should be set and that there should be clearly defined deliverables (p. 4). As in the previous example, the norms themselves employ guarded language and contain qualifiers, but it should be clear that the values underlying them (innovativeness and something like effectiveness and efficiency) pull in different directions. This is another instance of incommensurability: how does the value of adding innovative true beliefs compare to that of running a research project efficiently and effectively? It seems there is no more general value under which both of these could be subsumed to determine their comparative importance. Hence, it is not clear that there is a uniquely correct way to balance the two: it all depends on how much value one attaches to original and groundbreaking research versus realistic and manageable research. This is another case of incommensurability.

Incommensurability between norms means that there is *irreducible axiological pluralism* in codes of conduct. Why does this matter? For one thing, because incommensurability makes it impossible to compare the seriousness of integrity violations in a transparent systematic fashion and this, in turn, means that it is difficult to justify sanctions and their severity systematically and transparently. To illustrate, imagine an institutional research integrity committee that has to respond to two misconduct cases. In one case, a researcher changed the results under pressure from a sponsor and failed to disclose this. This clashes with epistemic norms about transparency and impartiality. In another case, a principal investigator repeatedly neglected her team on a major research project, which clashes with moral and professional norms about fair treatment of fellow researchers. Supposing the norms involved are indeed incommensurable, the committee has no way of comparing the severity of these two cases directly, and hence, it is very hard to say whether the same or different sanctions are justified in these two cases.

Note, however, that this is not to say that incommensurability between norms always makes justified decisions about what to do impossible. First, not all incommensurability leads to conflict. In contrast with the examples above, many incommensurable norms do not pull in different directions. For example, one can easily grant a Ph.D. student one is supervising authorship (thereby meeting a moral and professional norm) and report only correctly rounded *p* values in various studies (thereby meeting an epistemic norm). There is no conflict and no pressure to choose between the two, and this seems to apply to the majority of norms in most situations: they do not lead to incompatible prescriptions for how to act. Second, for particular conflicts between incommensurable norms, there might be good reasons for resolving them in a certain way even if there is no general way to compare these norms and the values underlying them. Suppose, for instance, that certain groups in society might be at a significant disadvantage. If it is foreseeable that a novel research project would negatively affect the wellbeing of these groups even further, this might be a good reason to give priority to a moral responsibility norm over an epistemic norm and thus to refrain from pursuing this project (see [29], pp. 93–108 for discussion). Such considerations, however, are heavily context-dependent and, therefore, cannot be captured in general rules as to how to weigh various incommensurable norms.

## Axiological pluralism 2: Conflicting norms from the same category

Let us now turn to conflicting norms within one category. Norms within a single category may or may not be incommensurable in the final analysis^14^, but it certainly looks like there are different norms even within one category, which can pull in different directions and lead to conflicting prescriptions for how to act. This presents another dimension of axiological pluralism. For the sake of brevity, we will only give examples of conflicting epistemic and moral norms.

### Conflicting epistemic norms

There are at least two core epistemic values that give rise to different and potentially conflicting epistemic norms. On the one hand, it is an epistemic value to *maximize true belief*. On the other hand, there is the epistemic value of *minimizing false belief*. These two values have been recognized since William James’ formulation of them.^15^

The following epistemic norms that we find in various codes arguably aim at maximization of true beliefs:

- The researcher must seek to offer an original and relevant contribution to advance science (Directives from Brazil, p. 21).
- Information will be presented truthfully and accurately in proposing, conducting, and reporting on research, and any claims made will be justifiable and evidence-based (Australian Code, p. 2).

We also find norms that seem primarily relevant to the avoidance of false belief:

- Authors and publishers consider negative results to be as valid as positive findings for publication and dissemination (ALLEA, p. 7);
- Research will be underpinned by attention to detail and robust methodology, avoiding or acknowledging biases (Australian Code, p. 2).

As in the previous section, we see that the examples of norms given here do not exemplify the two core epistemic values of maximizing true belief and minimizing false belief in their pure form. This is only to be expected, since we always have to balance two values, both in everyday life and in scientific practice. Even so, there is a case to be made that striving for novelty and originality mostly promotes the acquisition of true beliefs, whereas reporting negative results and acknowledging potential biases serves to avoid mistakes and so to minimize false beliefs.

To see that norms deriving from these two values can conflict, consider (unrealistically) that one maximizes true belief by simply believing everything, while minimizing false beliefs is accomplished by believing nothing. A more realistic conflict occurs, for instance, when the norm of presenting original and creative research clashes with the norm of *not* presenting (or avoiding) research that is biased. Imagine that one has proposed a new hypothesis and decides to put it to the test. One uses a new machine in the lab, but in the course of conducting the relevant experiments and measurements, the machine appears to suffer from minor malfunction. Upon technical inspection, however, it looks fine. The results are novel and original, but one has reason to think that they may be the product of one’s inexperience, programming errors, or some other unknown issue with the machine. In such a case, either one should present that research and be explicit about one’s suspicions about the machine’s malfunctioning, or one should refrain from publishing the study—at least for the time being, until one has more confidence in the results—even though it is novel and original. Inevitably, there will be scenarios in which norms regarding novelty and creativity favor publication and norms about carefulness count against (immediate) publication.

This shows that, even within the category of epistemic norms, we find norms promoting different epistemic values relevant to research integrity. Again, this need not always cause problems, but researchers should be aware that there will be situations in which they have to balance various norms.

### Conflicting moral norms

The moral realm, too, has potentially conflicting norms. An obvious example is the conflict between norms of openness on the one hand and norms of privacy and ownership on the other. In codes of conduct, we find a variety of epistemic, but also moral norms that encourage openness, transparency, and communication:

- Research methodology, research findings and knowledge will be shared and communicated openly (Australian Code, p. 2);
- Disclose and manage actual, potential, or perceived conflicts of interest (Australian Code, p. 4).

On the other hand, though, we find norms that advocate privacy, and, thus, *not* being open, transparent, or communicative about certain facts or data. For example:

- In performing a research project in collaboration with other researchers or as a member of a team, the researcher must maintain the confidentiality of all data, information, procedures, and partial results until the final results of the study are published, unless all collaborators and all team coordinators grant permission to disclose such information (Directives from Brazil, p. 22);
- The provisions for protecting the confidentiality of personal data, and respecting the privacy of subjects, including the precautions that are in place to prevent disclosure of the results of a subject’s genetic tests to immediate family relatives without the consent of the subject (Directives from Zimbabwe, p. 13).

From the point of view of openness and transparency, it would be best to reveal *all* data, so that everyone has access to it. From the point of view of privacy, though, it is sometimes best *not* to reveal all data.

## Overcoming pluralism?

At this point, one might wonder if the kinds of pluralism we have identified can be overcome. In other words, can we reduce certain norms to others (and mutatis mutandis the same for values and virtues)? A prior question, however, is why we would even want to overcome pluralism. What is wrong with it? After all, the normative scientific landscape that includes different kinds of values, norms, and virtues might simply be (highly) complex. We agree, and we will end up arguing that pluralism is inevitable. But that does not mean it is not useful to explore attempts to overcome pluralism. In general, simplicity is an important desideratum for scientific and philosophical theories, so it is worth exploring how much of it can be had here. Simplicity is also desirable for practical reasons: if we could overcome pluralism, it would be easier to rank various kinds of integrity breaches and to assign proper sanctions. Moreover, seeing how attempts to overcome pluralism fail can be instructive in appreciating the kind of pluralism we are dealing with even better.

One way to overcome plurality would be to develop an ordering of different sorts of norm violations based on a common measure for their seriousness. Below, we spell out and assess three attempts that even though they are meaningful and valuable for other purposes, do not solve the challenge of pluralism.

1. Determining the *prevalence* or *preventability* of different kinds of integrity breaches will not do the job. For by themselves, these measures tell us nothing about the absolute or relative seriousness of integrity breaches. Of course, if we were to have a measure of the seriousness of norm violations, information about their prevalence and preventability would be crucial for deciding what sorts of interventions for improving research integrity would be most promising, but that is a further issue beyond the scope of the present paper.
2. Factors such as whether a norm was violated intentionally or not and whether it was violated by an experienced scientist or by a freshly minted Ph.D. also do not provide a framework for comparing different kinds of norm violations. It is true that, other things being equal, the same violation done intentionally is more blameworthy than when done unintentionally. And it is true that the same violation is more blameworthy when done by an experienced researcher who should have known better, than when it is done by a fresh Ph.D. But, again, that implies nothing about how we should weigh the relative seriousness of, say, failing to grant authorship to a junior colleague compared to HARKing [28].
3. A more promising approach is that developed in Bouter et al. [8]. They rank about 60 questionable research practices in terms of what they label their “importance.” This is defined as frequency multiplied by impact, where impact is divided into two aspects: impact on trust in science and impact on scientific truth-finding. Both frequency and the two forms of impact are measured by expert estimates: scientists assigning values on a 5-point scale.^16^

This is indeed a step in the right direction if we are looking to make a systematic comparison and evaluation of various breaches of integrity. But there are at least three reasons why this approach does not overcome the forms of pluralism we identified above. (Note that this is not supposed to be a criticism of Bouter et al., for they never intended to use their measure to overcome pluralism.)

First, by focusing exclusively on trust impact and truth impact, the measure simply assumes a specific and contested normative moral theory, namely a form of consequentialism according to which only the consequences for trust and truth-finding determine the rightness or wrongness of academic (mis)behavior. This is a possible view, but it needs to be defended rather than assumed. To mention just two worries, some breaches of integrity may have no or only neglible impact on trust in science and truth-finding—for instance poor treatment of animals or very liberal granting of authorships—but we may nonetheless have good reason to discourage them. Moreover, two norm violations may have identical impact on trust and truth-finding, but we may have other grounds for deeming one more serious than another. One could, for instance, involve the breaking of a promise at significant professional cost to a junior colleague whereas the other is merely due to inattentiveness.

Second, a 5-point scale for trust and truth-finding impact is too crude a measure and obscures potential value conflicts from view. Suppose someone estimates that a certain breach of integrity has a great impact (say 5) on truth-finding. In light of our earlier discussion of the twin epistemic goal of maximizing true belief and minimizing false belief, it is unclear what this estimate means. That this breach causes scientists to avoid fewer false beliefs, to acquire less true beliefs, or both? Or, alternatively, that it causes scientists to do these things faster or slower? Depending on how one weighs these twin goals, one may be more serious than the other. Similar concerns arise for impact on trust: what does it mean when this is high? That science can be trusted less to discover truths, to treat scientists well, to take relevant social concerns into account, something else, all of these things?

Third, the fact that impact is measured by expert estimates is unsatisfactory. Scientists may not always have a clear view on how serious various breaches of integrity are. After all, estimating this requires, in addition to familiarity with the practice of science, normative analysis. This is not something scientists are especially equipped for. But even if we assume that scientists’ expert estimates can be trusted, the fact that they remain black boxes is unsatisfactory. As we argued above, there is strong reason to think that there are conflicting and sometimes incommensurable values underlying codes of conduct. Because of this, we should not rely blindly on (alleged) expert estimates. What we need is an explicit, systematic, and transparent account of how to weigh and compare the relevant norms, values, and virtues—or, if that is impossible in full generality, at least indications of how we can go about resolving cases of conflicting and incommensurable norms.

Hence, our conclusion stands: behind codes of conduct is an irreducible plurality of values that lead to different categories and kinds of norms. This realization should prompt intellectual humility in code drafters and code users. Because research integrity involves an irreducible plurality of sometimes incommensurable and conflicting norms of different kinds, one should assume that there will be multiple and equally reasonable ways of dealing with conflicts. Hence, it is important to listen to others, to transcend one’s default viewpoint in order to consider the merits of other points of view.^17^ Recently released codes attempt to guide judgments of which norms are more important by providing lists of assessment criteria when inspecting integrity breaches (see *Netherlands Code of Conduct for Research Integrity*, 2018, p. 24 or *Guide to Managing and Investigating Potential Breaches of the Australian Code for the Responsible Conduct of Research*, 2018). Such criteria are helpful, but they do not eliminate value pluralism. Rather, they identify considerations that should be factored into argumentations for why certain norms, values, or virtues are more important in a particular case than others. Justifying such argumentations requires an intellectually humble attitude, which is sensitive to both the plurality of norms, values, and virtues that constitute research integrity and to contextually relevant features of particular cases.

## Conclusions

We have shown the need for philosophical (namely, metaphysical and axiological) analysis of how various norms of research integrity and their breaches relate to each other. We have also provided some key ingredients for an axiological framework by arguing that (1) we ought to distinguish between norms, values, and virtues; (2) there are five relevant categories of norms: epistemic, moral, professional, social, and legal; (3) in some cases, norms from various categories and norms within the same category can conflict or be incommensurable; and (4) there is no common measure available to systematically compare different forms of research misconduct.

We would like to close with six recommendations for scholars working on research integrity:

1. In discussing issues in research integrity and in composing codes, we should distinguish explicitly between values, virtues, and norms, and account for how they take them to relate to each other.
2. In formulating norms, we should explicate what sort of norms we have in mind: epistemic, moral, professional, social, legal, or combinations thereof.
3. We should be aware that norms can be incommensurable with each other or conflict with each other in specific situations.
4. We should consider that even within the same category of norms, incommensurability or conflict is possible because different kinds of values underlie these norms.
5. In codes of research integrity, guidance should be given about what to do when norms are incommensurable or conflict with each other.
6. Scientists, scholars, and other code writers and code users should be intellectually humble in navigating conflicting norms and provide careful, context-sensitive reasons for the choice they make, as these choices may not be perfectly justified and sometimes not even perfectly justifiable.

## Data Availability

Not applicable

## Footnotes

1 For an example of that, see [41].

2 See [12, 40, 44, 45] for this.

3 We have intentionally not included the Singapore Statement on Research Integrity (https://www.wcrif.org/guidance/singapore-statement). This is because it is not itself a code of conduct but (successfully) serves as an international framework in which national codes can be developed. It does not specify standards or norms, but only contains more abstract values and virtues. We aim at the level of national codes that have the ambition to cover all disciplinary fields. There are many more detailed codes for specific disciplines. However, an analysis of value pluralism in these disciplinary codes falls outside the scope of our article.

4 This is a very rough take on the nature of values. The nature of values is central to the philosophical subdiscipline of value theory or axiology. Its central questions include the following: What is the exact nature of values? Are there objective values or are all values subjective, dependent on people’s preferences? What sort of values are there? Are some things intrinsically valuable, in and of themselves, or is all value instrumental? [34, 43] offer helpful overviews of key issues and contain many references to further literature.

5 In the actual text of the code, these things are designated as “fundamental principles,” but since they do not formulate rules or guidelines for behavior—which we take to be the hallmark of principles and rules—but describe fairly abstract qualities of actions and practices, we have included them under “values” here.

6 Sometimes, a distinction is drawn between *principles*, which are taken to more abstract, and *guidelines*, which translate abstract principles into more concrete rules. The distinction between more abstract and more concrete norms is obviously real, even though there is no sharp boundary between the two. But since this usage is not universal, we will continue to speak of abstract and concrete norms.

7 The exact relation between these two kinds of virtues is a subject of philosophical debate among virtue theorists. For our purposes here, it suffices if we can draw a prima facie plausible distinction between them.

8 ALLEA actually refers to these items as “principles.” Strictly speaking, this is a misnomer, as these terms are more naturally understood as designating personal qualities of a researcher or a research community. Cf. also note 6 above.

9 Although, as we said above, when values are characterized broadly, virtues are a subset of them. Strictly speaking, then, we would have only two kinds of things on our list here.

10 Attentive readers might wonder whether virtues, values, and norms all come in the five categories identified here. As this section will show, we think this is certainly the case for values and norms, but less clearly so for virtues. While philosophers commonly distinguish between moral and epistemic (or intellectual) virtues and sometimes talk about professional virtues, we are not aware of any work on social or legal virtues.

11 See [7, 25] for a detailed treatment of incommensurability and its relevance for practical reasoning and ethics.

12 These are two ways in which value pluralism is inevitable. This is not to deny that there might be further ways in which value pluralism is necessary. Moffatt [35] argues that there may be multiple but equally valid ways of knowing and multiple but equally valid accounts of scientific authorship. While this does not quite establish that there are irreducibly different values behind these different ways of knowing and accounts of authorship, Moffatt’s claims might be seen as congenial to the sort of pluralism we defend here.

13 Note that saying that two values are incommensurable does not mean that we cannot compare any norms that derive from them. In the present case, for instance, it is clear that we can compare the amount of disclosure prompted by a pure commitment to the epistemic value of openness with that prompted by pure commitment to honoring one’s contractual obligations. The same is true for any pair of incommensurable values: once we think about more concrete norms and behaviors that would promote these values, we will always be able to find some aspects in which these behaviors can be compared. But that does not mean we have found a fully general way of comparing the two values as such, which would allow us to resolve any conflict between them.

14 There are long-standing debates about this in both ethics and epistemology. Some consequentialists in ethics, for example, maintain that only pleasure is valuable (classically [6] [1789]), while others recognize multiple values, some of which are thought incommensurable [11]. In epistemology, some think truth is the only value [13], while others plump for value pluralism [2, 16].

15 See [27], p. 24. Many philosophers working in epistemology have accepted this as the twofold epistemic aim, e.g., [18], p. 317; [38], p. 55; [39].

16 Note that although Bouter et al. use this measure to evaluate the seriousness of a set of questionable research practices, it could be generalized to include evaluations of desirable research behavior. We could also estimate the truth and trust impact of following norms, engaging in good practices, etc.

17 See Baehr [4] for a discussion of the intellectual virtue of open-mindedness along these lines.
